# Between the Devil and the Deep Blue Sea—Resveratrol, Sulfotransferases and Sulfatases—A Long and Turbulent Journey from Intestinal Absorption to Target Cells

**DOI:** 10.3390/molecules28083297

**Published:** 2023-04-07

**Authors:** Izabela Szymkowiak, Malgorzata Kucinska, Marek Murias

**Affiliations:** 1 Curtis Health Caps S.A., ul. Batorowska 52, 62-081 Przeźmierowo, Poland; 2 Department of Toxicology, Poznan University of Medical Sciences, ul. Dojazd 30, 60-631 Poznan, Poland; 3 Center for Advanced Technology, Adam Mickiewicz University in Poznan, ul. Uniwersytetu Poznańskiego, 61-614 Poznan, Poland

**Keywords:** resveratrol, resveratrol sulfate, sulfotransferase, sulfatase, CYP450

## Abstract

For nearly 30 years, resveratrol has attracted the scientific community’s interest. This has happened thanks to the so-called French paradox, that is, the paradoxically low mortality from cardiovascular causes in the French population despite a diet rich in saturated fat. This phenomenon has been linked to the consumption of red wine, which contains a relatively high level of resveratrol. Currently, resveratrol is valued for its versatile, beneficial properties. Apart from its anti-atherosclerotic activity, resveratrol’s antioxidant and antitumor properties deserve attention. It was shown that resveratrol inhibits tumour growth at all three stages: initiation, promotion, and progression. Moreover, resveratrol delays the ageing process and has anti-inflammatory, antiviral, antibacterial, and phytoestrogenic properties. These favorable biological properties have been demonstrated in vitro and in vivo in animal and human models. Since the beginning of the research on resveratrol, its low bioavailability, mainly due to its rapid metabolism, especially the first-pass effect that leaves almost no free resveratrol in the peripheral circulation, has been indicated as a drawback that has hindered its use. The elucidation of such issues as pharmacokinetics, stability, and the biological activity of resveratrol metabolites is therefore crucial for understanding the biological activity of resveratrol. Second-phase metabolism enzymes are mainly involved in RSV metabolism, e.g., UDP-glucuronyl transferases and sulfotransferases. In the present paper, we took a closer look at the available data on the activity of resveratrol sulfate metabolites and the role of sulfatases in releasing active resveratrol in target cells.

## 1. Introduction

**Figure 1:**
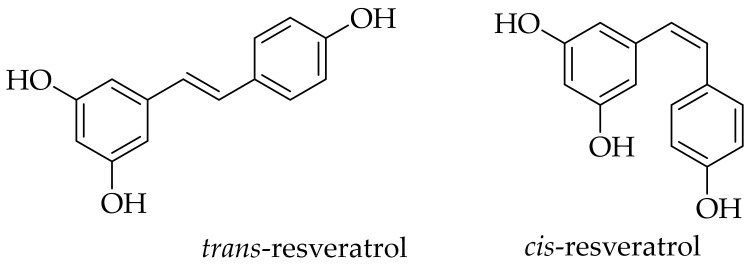
The chemical structures of the *trans* and *cis* isomers of resveratrol.

The scientific community’s interest in resveratrol (RSV) started from the discovery of the so-called French paradox: relatively low mortality from cardiovascular causes in the French population despite a diet rich in saturated fat. This phenomenon has been linked to the high consumption of red wine [1] and has been connected with resveratrol [2,3]. Currently, resveratrol is valued for its versatile, beneficial properties. Chemically, this compound is a stilbene derivative, namely 3,4′,5-trihydroxystilbene. It should be noted that stilbene is considered a privileged scaffold in medicinal chemistry [4]. Resveratrol exists in two isomers: *cis*-(Z) and *trans*-(E) (Figure 1). Initially, *cis*-resveratrol was thought to be biologically inactive. Today it is known that both isoforms possess biological activity. However, *trans*-resveratrol tends to be more potent [5] or, as proposed by Jhanji and co-workers, may exert different effects on the broad spectrum of molecular targets from distinct cellular pathways, e.g., tyrosyl-tRNA synthetase (TyrRS) [6]; histone serine-ADP-ribosylation [7]; NAD(P)H: quinone oxidoreductase-1 (NQO1) [8]; signal transducer and activator of transcription (STAT3) [9]; and many more, as excellently reviewed by Ko and co-workers [10]. The *trans*-isomer is also the most stable from the steric point of view [11]. Functionally, resveratrol is classified as a phytoalexin, a compound produced in plants as a defense mechanism in response to UV radiation, microbial infections, pesticides, and other factors [12].

**Figure 2:**
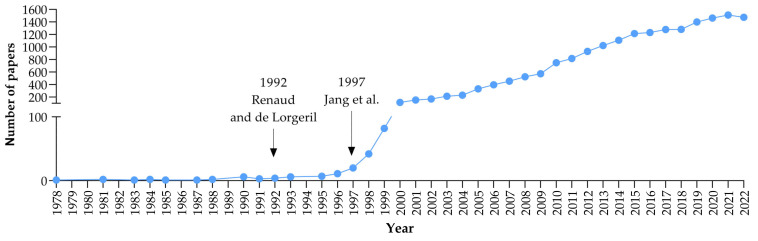
Resveratrol-related papers published in Pubmed.gov from 1978 to 2022, with significant acceleration visible after 1992 Renaud and de Lorgeril [3] and 1997—Jang et al. [16].

The first references to resveratrol in the literature come from the publication of Takaoka in 1939 on its isolation from the root of the white hellebore, *Veratrum grandiflorum* [13]. The name resveratrol comes from this source, as it is a resorcinol derivative of the *Veratrum* species [13]. However, it has been known for more than 2000 years in Ayurvedic preparations, such as darakchasava or manakka [14]. Resveratrol is produced naturally by 72 plant species, especially grapevines, pines, and legumes [2]. It is also in relatively high concentrations in mulberries, raspberries, pomegranates, peanuts, pistachios, and soybeans [2,5]. Another rich source of RSV is the root of *Polygonum cuspidatum*, used in traditional Chinese and Japanese medicine and called Hu-Chang and Ko-Yo-Kon, respectively [15]. Therefore, the French paradox theory and resveratrol’s cardioprotective effects were not the story’s beginning but only a turning point in modern history. It has become a viral topic among scientists because of its pleiotropic activity. At the end of the 20th century, two publications are believed to have caused an absolute avalanche of publications showing the beneficial effects of resveratrol. This was the work of Renaud and Longeril, indicating resveratrol as a factor responsible for the French paradox [3]. Moreover, a paper by Jang et al., summarises the knowledge of that time on the anticancer effects of resveratrol at all three stages of cancer development: initiation, promotion, and progression [16]. Later it was reported that resveratrol sensitizes cancer cells to chemotherapeutic drugs and reverses multidrug resistance if combined with clinically approved drugs [10]. Clinical trials in recent years showed this is a promising chemopreventive and therapeutic compound [10]. Each year up to 2021 has referenced a more significant number of publications that appeared in Pubmed.gov with resveratrol as a keyword. In the peak year of 2021, it was 1510 items; so, statistically, there were 4.1 publications in which the keyword resveratrol appeared daily. It seems this record will not be broken in 2022, as “only” 1473 papers appeared in Pubmed (Figure 2) in that year. 

**Figure 3:**
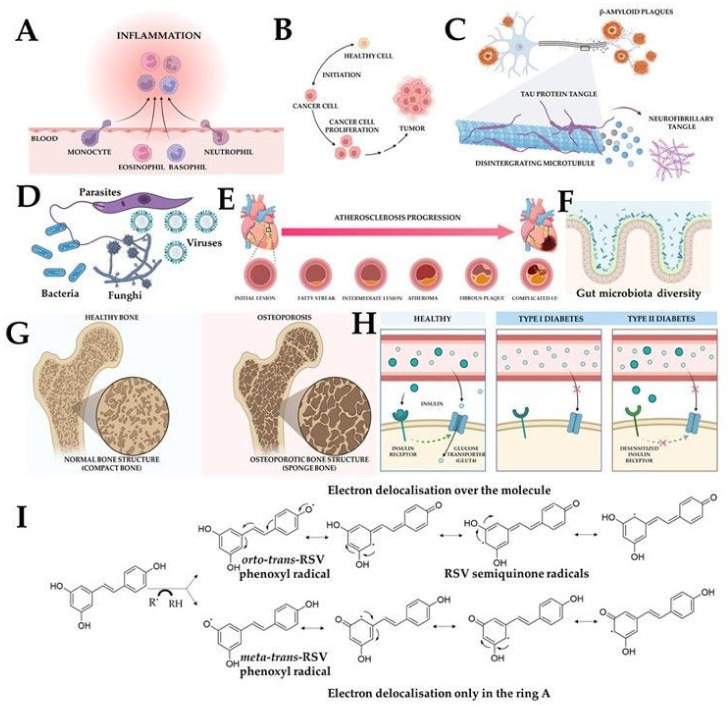
Pleiotropic activity of resveratrol molecule. Inflammation can be prevented by 3,4′,5-*trans*-trihydroxystilbene (**A**); it inhibits cancer development in its three primary stages: initiation, promotion and progression of cancer (**B**); neurodegenerative diseases (**C**). It has antiviral, antibacterial, antifungal, and antiparasitic activity (**D**). It can decrease the risk of atherosclerosis, and coronary heart diseases (**E**). Resveratrol modulates gut microbiota diversity to increase gut permeability and the integrity of intestinal tight junction proteins and modulates bone mineralisation and muscular metabolism (**F**). Increases bone mineral density and volume, decreases risk of osteoporosis (**G**) Moreover, it has protective effects against metabolic disorders (**H**) and can act as an antioxidant (**I**) (modified from [34]). Created with BioRender.com.

**Figure 4:**
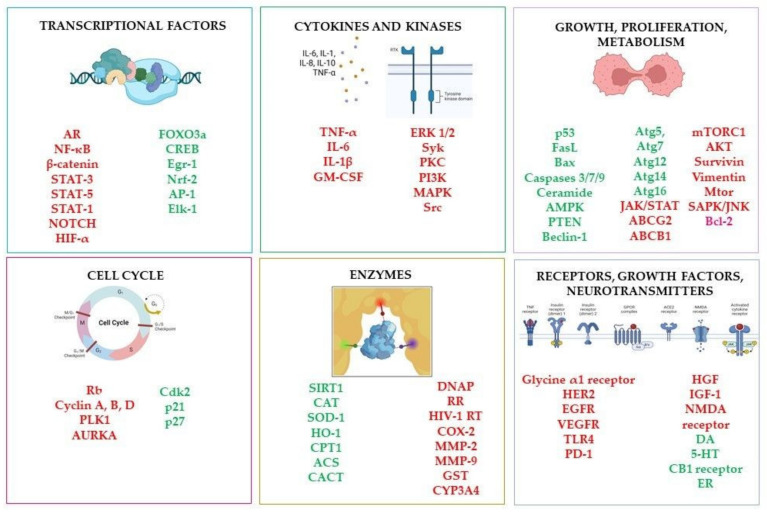
The selected molecular targets of resveratrol. The activated targets are marked in green, while the inhibited ones are in red. Created with BioRender.com. Abbreviations: AR—androgen receptor; ABCB1—ATP Binding Cassette Subfamily B Member 1; ABCG2—ATP Binding Cassette Subfamily G Member 2; ACS—Acetyl-CoA synthetase; AKT—Protein kinase B, serine/threonine-specific protein kinase; AMPK—AMP-activated protein kinase; AP-1—Activator Protein 1; Atg—anti-thymocyte globulin; AURKA—Aurora kinase A; Bax—Bcl-2-associated X protein; Blc-2—B-cell lymphoma 2; CACT—Carnitine-acylcarnitine translocase; CAT—Catalase; CB-1 receptor—Cannabinoid receptor type 1; Cdk2—Cyclin dependent kinase 2; COX-2—Cyclo-oxygenase 2; CPT-1—Carnitine palmitoyltransferase 1; CREB—cAMP-Response Element Binding protein; CYP3A4—Cytochrome P450 3A4; DA—Dopamine; DNAP—DNA polymerase; EGFR—Epidermal Growth Factor Receptor; Elk-1—ETS (E twenty-six) Like-1 protein; ER—Estrogen receptor ERK1/2—Mitogen-activated protein (MAP) kinase 1/2; FasL—Fas ligand; FOXO3a—Forkhead Box Protein O3a; GM-CSF—Granulocyte Macrophage Colony-Stimulating Factor; GST—Glutathione S-Transferase; HER2—human epidermal growth factor receptor 2; HGF—Hepatocyte growth factor; HIF-α—Hypoxia-Inducible Factor α; HIV-1 RT—HIV-1 reverse transcriptase; HO-1—Heme oxygenase-1; IGF-1—Insulin-like Growth Factor 1; IL-1β—Interleukin 1β; IL-6—Interleukin 6; JAK/STAT—Janus kinase-signal transducer and activator of transcription pathway; MAPK—Mitogen-Activated Protein Kinase; MMP-2—matrix metallopeptidase 2; MMP-9—matrix metallopeptidase 9; mTOR—Mammalian Target of Rapamycin; mTORC1—mammalian target of rapamycin complex 1; NFκB—Nuclear factor-kappa B; NMDA receptor—N-methyl-D-aspartate receptor; NOTCH—Notch signalling pathway receptor family; Nrf-2—Nuclear factor erythroid 2-related factor 2; p21—cyclin-dependent kinase inhibitor 1; p27—Cyclin-dependent kinase inhibitor 1B; p53—Tumor protein P53; PD-1—Programmed cell death 1 receptor; PI3K—Phosphoinositide 3-Kinase; PKC—Protein kinase C; PLK1—Polo-like kinase 1; PTEN—Phosphatase and tensin homolog deleted on chromosome 10; Rb—Retinoblastoma protein; RR—Ribonucleotide reductase; SAPK/JNK—stress-activated protein kinase/Jun N-terminal kinase; SIRT1—Sirtuin; SOD-1—Superoxide dismutase; Src—Non-Receptor Tyrosine Kinase; STAT—Signal Transducer and Activators of Transcription protein family; Syk—Spleen Associated Tyrosine Kinase; TLR4—Toll Like Receptor 4; TNF-α—Tumour Necrosis Factor α; VEGFR—Vascular Endothelial Growth Factor Receptor; 5-HT—Serotonine.

In these works, the impact on numerous cellular targets and cellular pathways by resveratrol, including the induction of apoptosisae (mainly via Bax, Caspase-3, Caspase-9, and Bcl-2 modulations) [17,18], cell cycle arrest [19] or retarding by vascular endothelial growth factor (VEGF) and metalloproteinase (MMP) inhibition [20,21] was described. Moreover, protection against free radicals was pointed out as a possible mechanism determining its beneficial activity. The antioxidant activity of resveratrol is associated not only with its ability to scavenge reactive oxygen species (ROS) [22]. Resveratrol also modulates cellular antioxidant defense through the transcription factor nuclear factor erythroid 2-related factor 2 and heme oxygenase-1 (Nrf2/HO-1) signalling pathway, increasing superoxide dismutase (SOD), catalase (CAT), glutathione peroxidase (GPx) activities and HO-1 protein levels, which are also connected to decreased lipid peroxidation [23,24]. Moreover, resveratrol reduces inflammation mainly via both forms of cyclooxygenase, nitric oxide (NO) and the NF-kB pathway, neuroprotection of cognitive and mental performance, aging deceleration, bone mineralization, and positive effects on metabolic syndrome [25,26]. RSV is also classified as a phytoestrogen; it interferes with estrogen receptors, and modulates pathways responsible for estrogen synthesis and metabolism [27,28]. Resveratrol also showed antimicrobial activity against various viral, bacterial, fungal, and parasitic species [29,30]. However, its low oral bioavailability casts a shadow over the therapeutic use of resveratrol [31,32,33]. The modulation of several biological processes by resveratrol is presented in Figure 3, while the most important molecular targets are shown in Figure 4.

Various solutions aimed at increasing the bioavailability of resveratrol have been under investigation in recent years [35,36]. For instance, the application of several bioactive compounds, such as quercetin or piperine, which inhibit sulfation or glucuronidation, have been tested [37,38,39,40]. Additionally, glucosylresveratrol and acylresveratrol [41], as well as conjugates of resveratrol with aspirin, have been proposed as solutions [40,41,42,43,44] In numerous studies, resveratrol has been administered in different formulations, including micronized powders and nanocarriers, to improve its bioavailability [25,31,37,39,41,42,44,45]. In the present paper, we discuss the available data on the activity of resveratrol sulfate metabolites.

## 2. Bioavailability of Resveratrol

**Figure 5:**
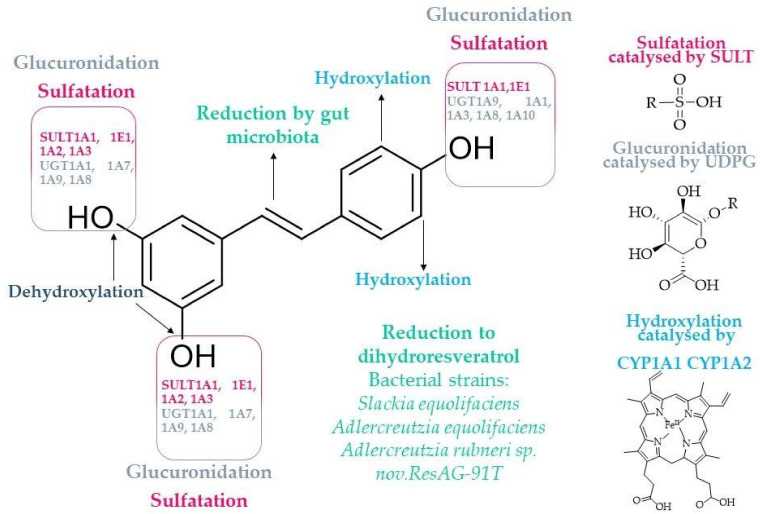
Main targets for drug-metabolizing enzymes in resveratrol molecule [28,60,65,66,68,69].

The explanation for the low bioavailability of resveratrol after oral administration (approximately 2%) lies in its absorption and disposition processes within an organism [33,46]. Resveratrol is a lipophilic compound; therefore, its absorption is affected by its low solubility in water (<0.05 mg/mL), which can be increased by the use of suitable solvents, including ethanol (50 mg/mL), and used in most of in vitro experiments dimethylsulfoxide (DMSO) (300 mg/mL) [11]. Absorption of resveratrol from the intestine occurs by passive diffusion and through transporters such as integrins, multidrug resistance-associated proteins (MRP2/ABCC2 and MRP3/ABCC3), or breast cancer-resistance protein (BCRP/ABCG2) [5,11,47,48,49]. Based on the total amount of metabolites excreted in the urine, it appears that the absorption of an oral dose of resveratrol is at least 70% [32]. Although resveratrol exists in free form in the bloodstream, glucuronide and sulfate metabolites are the majority [11]. The measurements of free resveratrol in blood in studies using a single dose of 25 mg resveratrol (equivalent to moderate red wine consumption) showed peak concentrations < 10 ng/mL (≈44 nM) 0.5-2 h after oral administration. Estimated plasma concentrations of resveratrol and its total metabolites were much higher, approximately 400–500 ng/mL (≈1.75–2.2 µM) [32,33]. The higher peak levels of resveratrol conjugates compared to the parent molecule may also suggest that they are responsible for some biological activity [5]. Approximately 90% of free *trans*-resveratrol binds to plasma lipoproteins due to its lipophilic nature [50]. Resveratrol also combines with albumin, a potential reservoir in the body, influencing its distribution and bioavailability simultaneously. In turn, complexes with lipoproteins and albumin can bind to specific receptors in the body’s cells and increase intracellular transport [11,51]. A mice study showed that resveratrol accumulates in tissues, and its volume of distribution (Vd) after a single intra-arterial dose is approximately 38 L/kg, indicating extensive extravascular distribution [52]. In human studies, the mean volume of distribution at a dose of 0.5 g was about 9 L, while for a 5 g dose was about 66 L [53]. This was despite resveratrol binding to serum albumin [54]. The human serum albumin-resveratrol complex formation and dissociation have been described in detail by Rezende et al. [55] and Cao et al. [56]. Studies in rats have shown an accumulation of resveratrol in the liver, heart, lungs, brain, and kidneys [57]. In contrast, studies in mice have shown accumulation in the intestine, heart, liver, lung, thymus and brain tissues [58,59,60]. Similar results of intestine accumulation were observed in studies on the human colon adenocarcinoma Caco-2 cell line [49]. The plasma half-life of free resveratrol varies in humans, ranging from 2 to 14 h [32,53,61]. The rapid metabolism of resveratrol also contributes to its low bioavailability. The process begins in the enterocytes and continues in the liver [62,63]. The main routes of metabolism are conjugation to sulfate and glucuronide residues catalysed by the phase II sulfotransferase isoenzymes SULT1A1, SULT1A2, SULT1A3, SULT1E1, and two isoforms of uridine 5’-diphospho-(UDP)-glucuronosyltransferases UGT1A1 and UGT2C7, respectively [15,32]. The most abundant metabolites in humans are: resveratrol-4′-sulfate, resveratrol-3-sulfate, resveratrol-4′-*O*-glucuronide [62,64]. The sulfation is considered the main conjugation pathway in humans and the limiting factor for the bioavailability of this compound [32]. On the other hand, rodents’ main metabolic pathway is glucuronidation. It is known that the formation of 3-sulfates occurs in the intestine and liver with *quasi*-exclusive participation of sulfotransferase SULT1A1 and only to a minor extent by SULTs 1A2, 1A3 and 1E1. At the same time, resveratrol-4′-*O*-sulfate is formed by SULT1A1, SULT1A2, and SULT1A2. The resveratrol-3-*O*-4′-*O*-disulfate formation is catalysed mainly by SULT1A1 [28,60,65]. It was also shown that SULT1A1 plays a crucial role in estrogen-dependent breast cancer cells and their resistance to treatment with resveratrol [28,66]. Moreover, SULT1A2, SULT1A3 and SULT1E1 were correlated with resveratrol-3-*O*-formation expression in breast tumour tissues and were significantly greater than in adjacent non-malignant tissue [66]. Some data also indicate the importance of pulmonary metabolism in resveratrol clearance. In human lung tissue, resveratrol is mainly conjugated with sulfate residues [67] (Figure 5).

Resveratrol is eliminated from the body through urine and faeces, with a varied recovery between 54% and 98% [46]. In a Phase I clinical trial, urinary excretion of resveratrol and its metabolites were rapid, with 77% of all metabolites derived from the compound excreted within 4 h of the 500 mg dose [53].

## 3. The Sulfate Metabolites

The biological activity of resveratrol demonstrated in in vitro studies is why it is regarded as a compound with promising therapeutic potential. However, due to the limitations in its bioavailability discussed above, evidence and mechanisms of in vivo efficacy are still under investigation. This phenomenon is called the *resveratrol paradox*, defined as the substance’s low bioavailability and high bioactivity [50]. Since plasma concentrations of resveratrol metabolites are around 20-fold higher [53] than those of the parent compound, they are considered to possess biological activity [31,70,71]. Plasma sulfate levels of 20–30 μM can be achievable in humans after repeated consumption of 1 g resveratrol per day [72]. The effects of resveratrol are mainly related to its 3-*O*-sulfate metabolite, which is the major metabolite in human plasma after oral administration [2,53,73]. Sulfates are also the primary metabolites in Caco-2 cells; however, resveratrol may act as a sulfatation inhibitor [62]. The half-life of resveratrol sulfate conjugates in plasma range from 3.2 to 11.5 h [53]. Alternatively, sulfate metabolites may be reconverted into the parent molecule at the target organs and thus contribute significantly to in vivo efficacy by providing a more stable, transient form of the compound [72,74].

### 3.1. Anticancer Activity

The chemopreventive potential of resveratrol is one of its most widely studied biological activities [75]. Resveratrol influences the process of carcinogenesis at the three stages: tumour initiation, promotion, and progression [17]. It can activate apoptosis, arrest the cell cycle, or inhibit kinase pathways. Moreover, resveratrol’s blood levels seem sufficient to act as an anti-invasive agent [76,77]. Several of these activities have also been confirmed for resveratrol sulfate conjugates. These effects are due to several mechanisms related to the inflammatory response’s down-regulation [78]. This includes inhibition of the synthesis and release of pro-inflammatory mediators, modification of eicosanoid synthesis, inhibition of immune cell activation, inducible nitric oxide synthase (iNOS), cyclooxygenase-2 (COX-2), and modulation of NF-κB [70].

Resveratrol-3- and 4′-*O*-sulfates, when administered to mice, were absorbed and hydrolysed, releasing free resveratrol in plasma and tissues. The intracellular hydrolysis of monosulfate metabolites to resveratrol was confirmed in a panel of human colorectal and breast tissue cell lines. The degree of uptake dictated the antiproliferative activity and depended on the presence of specific membrane transporters. Therefore, the potential exists for prolonged intracellular regeneration of the parent compound within internal target tissues for as long as these conjugates persist, which is at least 24 h for the 3-*O*-sulfate in human plasma. Pharmacologically achievable concentrations of sulfate metabolites induced autophagy and senescence in cancer cells, which has broad implications for treating various chronic and aging-related diseases. Concentrations in the range of 25 μM inhibit the proliferation of HT-29 and HCA-7 cancer cells by about 20%, while sparing HCEC cells derived from the normal epithelium. Furthermore, concentrations achieved in human colorectal tissue averaged 50 μM but could reach up to about 640 μM, which exceeds the concentrations required for 95% inhibition of HT-29 cell growth (250 μM) [72].

The disturbed activity of the NF-κB pathway may promote cancer progression and other non-oncological diseases [79]. The research of Hoshino et al. on human embryonic kidney cell line 293/NF-κB-Luc has revealed 4′-*O*-sulfate as the most active metabolite in inhibiting NF-κB signalling pathway [70]. The chemopreventive activity of resveratrol may be partly due to the quenching of unstable free radicals and reducing DNA damage by ROS [80]. The presence of hydroxyl groups in the molecule is responsible for this activity. Hoshino et al. tested the antioxidant properties of resveratrol sulfate metabolites by measuring their ability to quench the 2,2-diphenyl-1-picrylhydrazyl (DPPH) radical. While the activity of resveratrol potassium 3-sulfate (with remaining hydroxyl groups at a position of 5, and 4′) was comparable to the parental compound, resveratrol potassium 4′-sulfate was slightly lower [70]. The metabolites without any free hydroxyl groups were inactive as ROS scavengers.

Induction of NAD(P)H: quinone reductase 1 (QR1) is a well-studied mechanism of cancer chemoprevention. This process usually coincides with the induction of another phase II detoxification enzyme. The metabolite 3-*O*-Sulfate was more potent than resveratrol in the rapid and sensitive QR1 cellular assay, while the other sulfate metabolites were less potent. However, they all showed some degree of activity [70]. Patel et al. found that patients with confirmed colorectal cancer who received 500 mg and 1000 mg of resveratrol daily for eight days before surgery had high levels of conjugates in the colon, mainly resveratrol-3-*O*-sulfate and two glucuronides [81]. Moreover, the cellular uptake of resveratrol sulfate metabolites into the cytoplasm of tested human colon cancer cells was cell-line dependent (HCEG < HCA-7 < HT-29). These results suggested the uptake by active transport instead of passive diffusion. Their results indicated that organic anion-transporting polypeptide 1B3 (OATP1B3) is involved in transporting resveratrol sulfates [72]. In another study employing colon cancer cell lines, Aires et al. showed that resveratrol metabolites, especially resveratrol-3-*O*-sulfate, inhibit the proliferation of SW480 adenocarcinoma cells and their SW620 metastatic progenitor cells, which was as a result of cell cycle arrest in the S phase. They also showed that mixtures of three resveratrol metabolites (resveratrol-3-*O*-sulfate, resveratrol-3-*O*-glucuronide and resveratrol-4′-*O*-glucuronide) induced antiproliferative activity with greater than resveratrol or its metabolites in monotherapy. The antiproliferative effect was accompanied by a cell cycle blockade in the S phase and DNA damage induction, resulting in apoptotic cell death. All these processes enhanced the anti-tumour activity of SN38 (irinotecan’s active metabolite) and oxaliplatin in metastatic SW620 tumour cells [82].

**Figure 6:**
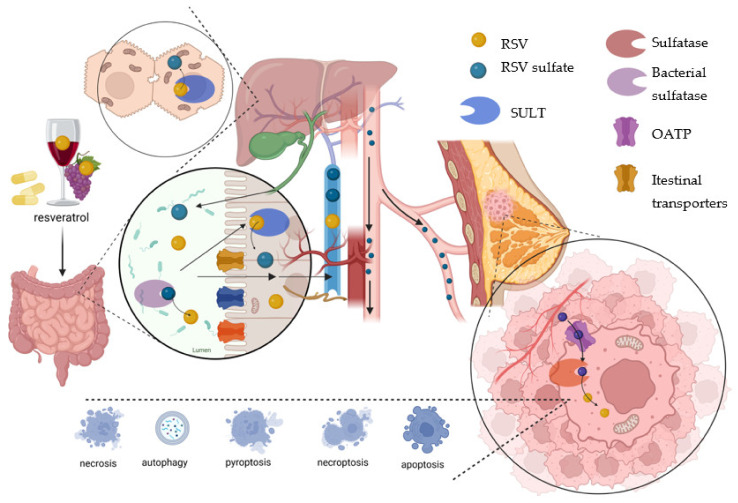
Long and turbulent journey of resveratrol from the ingestion to the gastrointestinal tract, via the gut wall, following sulfation and glucuronidation to the bloodstream. First-pass effect in the liver (sulfation and glucuronidation) and transport to organs, tissues, and cell uptake is possible. For instance, in breast cancer cells, OATP carriages resveratrol sulfate to cancer cells, while cleavage by sulfatases is possible. In this process, native resveratrol is released and may exert its anticancer effect inducing cell death (apoptosis, autophagy pyroptosis, necroptosis, necrosis). On the other hand, the passage from the liver via bile to intestinal and cleavage resveratrol sulfate to resveratrol to resveratrol by gut microbiota is also possible. Created with BioRender.com.

Indeed, organic anion-transporting polypeptides (OATPs) mediate the uptake of many clinically significant drugs. Riha et al. investigated their role in the cellular transport of resveratrol and its primary metabolites. This study was performed in OATP-expressing Chinese hamster ovary (CHO) cells and estrogen-dependent breast cancer ZR-75-1 cells. Resveratrol-3-*O*-sulfate was exclusively transported with a low affinity by OATP1B3, which should be considered in humans as the leading player after oral intake of dietary resveratrol. Among the 11 human OATPs, OATP1B1 and OATP1B3 are highly expressed in the liver and mediate the uptake of numerous drugs into hepatocytes [83]. They showed that resveratrol, resveratrol 3-*O*-sulfate, and resveratrol 3-*O*-4′-*O*-disulfate are substrates for these OATPs, while resveratrol glucuronides and resveratrol-4′-sulfate were not substrates for OATPs. In the ZR-75-1 and OATP knockdown ZR-75-1 cell culture model, they proved that OATP-dependent transport of sulfate to these cells, together with the high activity of intracellular sulfatases, led to rapid deconjugation of sulfates into pharmacologically active resveratrol. This process might be responsible for the observed pharmacological activity of resveratrol. The Authors indicated that future in vivo studies should focus on the concentration of resveratrol and its conjugates in target tissues on the levels of OATP and sulfatase expression [83] (Figure 6).

### 3.2. The Sulfate Metabolites’ Anti-Inflammatory Activity

In terms of anti-inflammatory activity, sulfate conjugates modulated inflammatory pathways in vitro, and in some cases, their efficacy, is similar to that of the parent compound [70]. Cardio- and chemoprotective effects of resveratrol relate to COX-1 and COX-2 inhibition. These enzymes are responsible for converting arachidonic acid into prostaglandins and other eicosanoids. It is well known that these metabolites can stimulate tumour growth in humans and experimental animals [70,71]. Resveratrol and its 4′-*O*-sulfate metabolite are potent inhibitors of both cyclooxygenase isoforms (IC_50_ values are presented in Table 1) [70,71]. The concentrations of resveratrol and 4′-*O*-sulfate conjugate in human plasma after oral administration of the resveratrol were 0.5 μM and 2 μM to 10 μM, respectively [53]. Knowing the activity of these isoforms, effective in vivo inhibition of both COX-1 and COX-2 was possible. Furthermore, resveratrol binds exclusively to the COX active site. The hydroxyl groups stabilise this binding, which allows the exclusion of arachidonic acid binding and thus inhibits catalysis. Resveratrol binding occurs like that observed with popular non-steroidal anti-inflammatory drugs (NSAIDs), such as ibuprofen, diclofenac or aspirin [71,78].

Physiologically, nitric oxide is a signalling molecule in various processes, such as vasodilation and the immune response. However, prolonged and excessive NO production by inducible nitric oxide synthase (iNOS) promotes pathologies such as chronic inflammatory diseases and cancer. Furthermore, increased NO synthesis may contribute to tumour growth by promoting angiogenesis. Inhibitors of iNOS, including resveratrol, may have chemopreventive effects due to their antiproliferative activity. It was determined that at an IC_50_ value of 15 μM, resveratrol was the most potent inhibitor of iNOS. The 4′-*O*-sulfate and 3-*O*-sulfate, and other derivatives possessed moderate activity [70]. However, in the Ladurner et al. study, none of the resveratrol metabolites tested, including resveratrol-3-*O*-sulfate and resveratrol-4′-*O*-sulfate, significantly increased eNOS enzyme activity and endothelial NO release or affected intracellular ROS levels [84].

### 3.3. Anti-Aging Effect

Another effect of resveratrol is the slowing of the aging process. Several reports indicated that resveratrol acted as a life-extending agent. It was shown for yeast, nematodes (*Caenorhabditis elegans)* [85], mice (including HtrA2 knockout mice), fruit flies (*Drosophila melanogaster)* [86], honey bees (*Apis mellifera*) [87], and turquoise killifish achieved by activating human NAD^+^-dependent deacetylating protein 1 (SIRT1). Incubation of sulfated metabolites with SIRT1 showed the same stimulation of the enzyme activity as observed with resveratrol. In the analyses by Calamini et al., the values of the apparent activator constants (Ka) for resveratrol and its sulfate metabolites were similar. This indicates that both sulfate analogues can activate SIRT1 to the same extent as resveratrol. These data suggest that resveratrol and its metabolites have an analogous mechanism of action when measured employing lysyl deacetylase activity [71].

**Table 1:** The basic biochemical parameters of resveratrol and its sulfates, indicating their cancer chemopreventive activity.

Assay	Resveratrol	Resveratrol-3-*O*-4′-*O*-Sulfate	Resveratrol-3-*O*-Sulfate	Ref.
COX-1 inhibition at 34 µM (%)	75.2 ± 4.53	63.2 ± 3.39	74.3 ± 0.99	[70]
COX-2 inhibition at 34 µM (%)	72.2 ± 4.67	65.8 ± 7.64	62.0 ± 1.7	[70]
NF-κB inhibition (%)	75.7 ± 2.12	64.0 ± 2.26	33.0 ± 4.81	[70]
Nitric Oxide inhibition (%)	71.8 ± 3.5	56.8 ± 5.9	41.0 ± 0.7	[70]
Aromatase inhibition (%)	34.8 ± 1.21	30.4 ± 0.56	28.2 ± 1.12	[70]
DPPH inhibition (%)	65.2 ± 2.0	42.8 ± 2.5	68.0 ± 1.9	[70]
Cytotoxicity KB cells survival at 20 µg/mL (%)	47.8 ± 5.2	70.3 ± 8.8	108.5 ± 10.3	[70]
Cytotoxicity MCF-7 cells survival at 20 µg/mL (%)	38.6 ± 3.5	103.4 ± 8.8	51.7 ± 0.2	[70]
COX-1 IC_50_ (µM)	6.65 ± 2.5	5.55 ± 1.73	3.60 ± 0.8	[70]
COX-1 IC_50_ (µM)	1.1 ± 0.44	5.1 ± 0.55	68 ± 2.8	[71]
COX-2 IC_50_ (µM)	0.75	8.95	7.53	[70]
COX-2 IC_50_ (µM)	1.3 ± 0.40	2.5 ± 0.35	>300	[71]
Nitric Oxide IC_50_ (µM)	0.173 ± 0.05	18.2 ± 0.99	-	[70]
IC_50_ (µM) RAW 264.7 cells	15.0 ± 2.6			[70]
DPPH IC_50_ (µM)	178.5 ± 9.3	-	219.2 ± 3.1	[70]
Estrogenic activity (yeast) EC_50_ hERα (M)	-	-	3.80 × 10^−4^ ± 0.01	[88]
Estrogenic activity (yeast) EC_50_ hERβ (M)	-	-	2.20 × 10^−4^ ± 0.05	[88]
QR1CD (µM)	21 ± 0.46	>6.9	2.6 ± 0.38	[70]
SIRT1 Ka (µM)	32.2 ± 3.4	36.4 ± 6.7	52.6 ± 6.6	[71]

EC_50_ indicates the molar compound concentration causing half-maximal induction of the β-galactosidase reporter.

### 3.4. Delipidating Activity

Resveratrol also has beneficial effects on diseases associated with metabolic syndrome and obesity. Lasa et al. studied the effects of resveratrol and its metabolites on 3T3-L1 adipocytes [89]. They showed that both resveratrol and *trans*-resveratrol-3-*O*-sulfate significantly reduced the triacylglycerol content in maturing pre-adipocytes (−13.0% and −20.0%, respectively). However, this sulfate metabolite did not affect mature adipocytes. Moreover, it reduced CCAAT-enhancer-binding protein α (C/EBP-α), peroxisome proliferator-activated receptor γ (PPAR-γ), and lipoprotein lipase (LPL) expression. All are known as the transcriptional factors involved in adipogenesis [89]. The same team, in another study, investigated the effect of resveratrol metabolites on the expression and secretion of adipokines. Adipokines, produced by adipose tissue, can act as autocrine or paracrine modulators, modifying metabolic pathways related to lipid metabolism and glucose homeostasis [90,91]. A vast number of adipokines were identified, including leptin, adiponectin, C1q/ tumour necrosis factor (TNF) related proteins (CTRPs), visfatin, vaspin, apelin, chemerin, and omentin [92,93]. Leptin is related to glycemic control. A physiological increase in plasma leptin inhibits insulin secretion [94]. Additionally, visfatin and apelin are partially involved in glucose homeostasis; consequently, they are tangled in mechanisms responsible for the development of obesity. Apelin increases energy expenditure and upregulates the expression of uncoupling proteins UCP1 and 3; moreover, it reduces the respiratory quotient, increasing fat oxidation. Both visfatin and apelin improve insulin sensitivity and maintain glucose homeostasis. Furthermore, visfatin is essential in pancreatic β-cells as an intra- and an extracellular NAD biosynthetic enzyme. It is also involved in glucose-stimulated insulin secretion. On the other hand, apelin enhances glucose uptake in insulin-responsive tissues, e.g., skeletal muscles [95,96]. Resveratrol-3-*O*-sulfate reduced leptin mRNA and increased apelins and visfatin, while resveratrol also decreased leptin mRNA but increased adiponectin. It could be suggested, therefore, that resveratrol metabolites play a part in these beneficial effects of resveratrol [95].

### 3.5. Antiestrogenic Activity

Aromatase, also known as CYP19A1, is an enzyme converting androgens to estrogens by hydroxylation. Its transcription is mediated by IκB kinase (IKKβ), previously known for its tumour-promoting activity, and its inhibitors, e.g., resveratrol, have been shown to act as a chemopreventive agent [97,98]. In vitro, resveratrol and its sulfates appeared to be relatively weak aromatase inhibitors. The parent compound inhibited the enzyme activity in less than 35% and was the most active of the metabolites-4′-sulfate 30% at a concentration of 34 μM [70]. Furthermore, due to its structural similarity to estradiol, resveratrol interacts with human estrogen receptors (hERs) on both estrogen receptor α (hERα) and estrogen receptor β (hERβ). Therefore, it is classified as a phytoestrogen. It is assumed that resveratrol acts as a mixed agonist/antagonist at low concentrations, whereas at higher concentrations and in the presence of 17-β-estradiol, it behaves as a pure antagonist. As shown in the study by Ruotolo et al. using a yeast two-hybrid (Y2H) assay involving ligand-dependent recruitment of a transcription coactivator by either form of hER, only resveratrol-3-*O*-sulfate exerted an apparent anti-estrogenic effect on both hER receptors [88]. The marked preference for hERα was confirmed in an hERα-positive MCF-7 human breast adenocarcinoma cell line. In contrast, resveratrol-3-*O*-sulfate proved to be an extremely weak and non-selective antagonist of hERα. Furthermore, in the cited study, resveratrol-3-*O*-sulfate reached an estimated intracellular concentration of 60 nM, which, although 10-fold lower than the more lipophilic resveratrol, was sufficient to induce an approximately 50% reduction in 17-β-estradiol activity [88].

### 3.6. Modulating the Integrity of the Tight Junction Barrier and the Composition of Intestinal Microflora

Studies of the effects of resveratrol and its metabolites in modulating the integrity of the tight junction barrier showed a significant upregulation of transepithelial electrical resistance (TEER) in Caco-2 cells incubated with resveratrol and resveratrol-3-*O*-sulfate at a concentration of 100 μM. The mRNA expression of the genes occludin, zonula occludens-1 (ZO-1), and claudin-4, associated with forming intestinal tight junctions, was significantly upregulated after resveratrol-3-*O*-sulfate administration compared to the control. Furthermore, resveratrol-3-*O*-sulfate induced a more substantial up-regulation of occludin and claudin-1 gene expression than resveratrol. The significant components of the intestinal mucosal structure are also mucins (MUC), including MUC1, MUC2, and MUC3, which facilitate epithelial protection. Zhang and coworkers showed that treatment with resveratrol-3-*O*-sulfate significantly increased the expression of MUC3 compared to controls [99]. Resveratrol-3-*O*-sulfate was present in both intestines and significantly increased the expression of tight junction proteins and mRNA of mucins. Resveratrol-3-*O*-sulfate exerted more potent beneficial effects than the parent compound.

Based on these results, resveratrol-3-*O*-sulfate was considered a critical functional component of resveratrol in regulating gut barrier function in vivo. The same group showed that resveratrol sulfate conjugates, specifically resveratrol 3-*O*-sulfate, demonstrated a positive effect on the composition of intestinal microflora as a result of selectively inhibiting or promoting the growth of bacterial abundance. This compound, in contrast to the parent compound, had a significantly beneficial effect on the increase in the growth of *Lactobacillus* species. One of them is the *Lactobacillus reuteri* strain (approximately three-fold), a probiotic that regulates metabolic syndrome and intestinal immunity, and inhibits pathogen infections [99,100]. Studies published a few years earlier showed that resveratrol increased the relative abundance of *Lactobacillus* and *Bifidobacterium* strains in mouse models of colitis and obesity [101,102]. These data suggest that the regulation of intestinal microbes by resveratrol in vivo is mainly mediated by the potency of its intestinal metabolites [99]. Moreover, studies showed a decrease in two *Bacteroides* species after in vitro fermentation with resveratrol-3-*O*-sulfate. Similar results were obtained when the combination of epigallocatechin-3-gallate and resveratrol was applied to overweight women and men [99,103]. Another study using resveratrol in vivo showed increased *Bacteroides* [104]. The differences may be because polyphenols can regulate the gastrointestinal antimicrobial response produced by the host and the gut microbiota [105]. For example, the host’s secretion of IgA and antimicrobial peptides and the secretion of bacteriocins and antimicrobial peptides by the gut microbiota can regulate the gut flora [99]. Many authors mention that resveratrol metabolism in gut microbiota shows interindividual differences and depends strongly on the microbiome composition. Yao et al. showed that the presence of the probiotic strain *Ligilactobacillus salivarious* enhances the conversion of resveratrol to dihydroresveratrol [106]. The same activity was also confirmed for other bacterial strains from the human intestinal microbiota, including *Slackia equolifaciens*, *Adlercreutzia equolifaciens*, and *Eggerthella lenta* ATCC 43055 [69]. Moreover, there are reports of the recycling of resveratrol, which involves the restoration of the parent compound from bile-secreted metabolites back into the intestinal lumen following its reabsorption [107]. This is demonstrated by a second peak in plasma concentration at 6–8 h after oral administration [32,46,53,108]. The hydrolysis of conjugates is also thought to occur in target tissues. Therefore, metabolites circulating in the bloodstream provide a reservoir of the compound in the body [31,109]. It may be mentioned also that the gut microflora also plays a crucial role in the intestinal metabolism of resveratrol, specifically in the hydrolysis of glucuronide conjugates [99]. This activity results in the restoring of the original form of resveratrol before the second transformation to dihydroresveratrol, lunularin, 3,4′-dihydroxy-*trans*-stilbene, piceid and their glucuronides and sulfates [62,110].

### 3.7. In Silico Calculations

Currently, more and more programs that allow in silico calculations of topological descriptors and based on them, ADMET (absorption distribution metabolism elimination, toxicology) properties and various aspects of toxicity, are available on the Internet free of charge. Resveratrol and its metabolites have been saved in the form of smiles notation and entered in the interface of SwissAdme, PreADMET, admetSAR, and ProTox-II freely available web servers. The parameters obtained were assigned to groups presenting: ADME properties, toxicological parameters, and topological descriptors. They are shown in Table 2, Table 3 and Table 4, respectively.

The biological activity of xenobiotics depends mainly on the processes referred to as ADME (absorption distribution metabolism elimination). Interactions of xenobiotics with enzymes responsible for their distribution (e.g., transmembrane transporters) and their metabolism (e.g., drug metabolism enzymes) were predicted and presented in Table 2.

Moreover, prediction results of possible interactions with key drug-metabolizing enzymes, transmembrane transporters, and receptors are presented. Calculations were made using four programs. Some of this software allows the evaluation of the same parameters. The predictions obtained were compared in terms of their mutual agreement. It should be admitted that the programs used for the calculations gave consistent results for resveratrol, but in some cases, they provided different results for their metabolites. Contradictory results were obtained, e.g., for monosulfates as inhibitors of CYP2C19 and CYP3A4. The inhibition of CYP2C19 was experimentally evaluated by Seki and co-workers, who showed that resveratrol might reduce CYP2C19 activity in a preincubation time-dependent manner [115]. As expected, in most cases, after adding at least one sulfate group, the metabolites lost their activity as inhibitors or substrates for xenobiotic-metabolizing enzymes or membrane transporters. However, conflicting results were obtained for the estrogen and androgen receptors. Similarly, contradictory information was received when crossing the blood-brain barrier had been predicted. On the other hand, the data on foreseeing the ability to transport resveratrol by glycoprotein p (P-gp), the ability to inhibit it was perfectly consistent.

A set of parameters describing various aspects of the toxicity of resveratrol and its sulfated metabolites is presented in Table 3.

As expected in most cases, resveratrol sulfates have weaker biological activity than resveratrol. Simulations suggest that sulfates do not cause loss of mitochondrial membrane potential, which usually coincides with the opening of the mitochondrial permeability transition pores, leading to the release of cytochrome c into the cytosol. According to performed predictions, resveratrol may cause eye irritation and skin sensitisation and cause fish and Crustacea toxicity. Interestingly, according to the results obtained *in silico*, resveratrol may exert a mutagenic effect in the Ames test [113]; what is more, similar results may be obtained for resveratrol-3-*O*-sulfate [113]. In contrast, resveratrol-4′-*O*-sulfate and resveratrol-4′-*O*-sulfate should have a negative impact, according to simulations in these programs. The best-known drug likeness rule is the Lipinski rule of five [116,117] which was estimated in this study using the SwissADME web tool [111] (version 2.4, Molecular Modelling Group, Swiss Institute of Bioinformatics, Lausanne, Switzerland). This is a set of guidelines developed at Pfizer and used to predict the oral bioavailability of a potential drug candidate. Although it is not a definitive measure of oral bioavailability, many compounds violate one or more criteria but are well absorbed from the gastrointestinal tract. The law states that a compound is more likely to be orally bioavailable if it fulfils the following criteria: molecular weight less than 500 Daltons; LogP less than five; fewer than ten hydrogen bond donors, fewer than five hydrogen bond acceptors. Like Lipinski’s rule of five, all tested compounds passed Ghose (developed at the Indian Institute of Technology, Delhi) [118], a rule-based algorithm to predict the drug-likeness of small molecule compounds. The compounds that are likely to be promising candidates for further development should pass the following criteria: 160 ≤ molecular weight ≤ 480; 0.4 ≤WLOGP ≤5.6; 40 ≤ MR ≤ 130; 20 ≤ number of atoms ≤ 70. The Veber rule (developed at GlaxoSmithKline) is based on only two structural and physic-chemical properties: the number of rotatable bonds (should be less than 10) and polar surface area (should be less than 140 Å^2^) [119]. The Egan rule from Pharmacopeia Inc evaluates WLogP, which should not exceed 5.88, and TPSA, which should not be larger than 131.6 Å^2^ [120]. According to the Muegge filter from Bayer, compounds with good oral bioavailability should meet the following criteria: 200 ≤ Molecular Weight 600; −0.2 ≤ XLOGP ≤ 5, TPSA≤150, number of rings ≤ 7; the number of carbons > 4; numbers of heteroatoms >1; the number of rotatable bonds ≤ 15; the number of H-bond acceptors ≤ 10; the number of H-bond donors ≤ 5 [121]. The Veber, Egan and Muegger rules were violated only by resveratrol-3-*O*-4′-*O*-sulfate since its TPSA is 164.19Å^2^ (Table 4). The relatively simple leadlikeness rule brings one violation for resveratrol and one for resveratrol-3-*O*-4′-*O*-sulfate, since their molecular weights are outside the range of 250 and 350, respectively. According to this rule, XLogP should be ≤5, while the number of rotatable bonds ≤ 7 [122]. No alerts were generated from Pan Assay Interference Structures (PAINS) described by Beall and Holloway [123]. At the same time, stilbene scaffold and sulfonic acid were indicated as “alert” structures according to rules described by Brenk and co-workers [124]. The synthetic accessibility has been assessed as 2.02 for resveratrol, followed by 2.71 for resveratrol-4′-*O*-sulfate, 2.84 for resveratrol-3-*O*-sulfate, while resveratrol-4′-*O*-sulfate accessibility has been scored as 3.01. where 1 is very easy, and 10 is difficult [111] (Table 4). For a long time, however, synthesising resveratrol sulfates has been considered challenging, as reflected in the titles of papers describing these procedures [70,125].

**Table 4:** The basic topological parameters of resveratrol and its sulfates are calculated using free web-based programs. Reference [111] corresponds to SwissADME.

Assay	Resveratrol	Resveratrol-4′-*O*-Sulfate	Resveratrol-3-*O*-Sulfate	Resveratrol-3-*O*-4′-*O*-Sulfate	Ref.
Formula	C_14_H_12_O_3_	C_14_H_12_O_6_S	C_14_H_12_O_6_S	C_14_H_12_O_9_S_2_	[111]
MW	228.24	308.31	308.31	388.37	[111]
#Rotatable bonds	2	4	4	6	[111]
#H-bond acceptors	3	6	6	9	[111]
#H-bond donors	3	3	3	3	[111]
MR Molar refractivity	67.88	78.08	78.08	88.28	[111]
Topological Polar Surface Area (TPSA) (Å^2^)	60.69	112.44	112.44	164.19	[111]
iLOGP	1.71	1.25	0.69	0.50	[111]
XLOGP3	3.13	2.53	2.53	1.94	[111]
WLOGP	2.76	3.31	3.31	3.87	[111]
MLOGP	2.26	1.64	1.64	1.16	[111]
Silicos-IT Log P	2.57	0.94	0.94	−0.62	[111]
Consensus Log P	2.48	1.94	1.82	1.37	[111]
ESOL Log S	−3.62	−3.50	−3.50	−3.43	[111]
Ali Log S	−4.07	−4.54	−4.54	−5.01	[111]
Silicos-IT LogSw	−3.29	−2.93	−2.93	−2.54	[111]
ESOL Solubility (mg/mL)	5.51 × 10^−2^	9.65 × 10^−2^	9.65 × 10^−2^	1.45 × 10^−1^	[111]
Ali Solubility (mg/mL)	1.93 × 10^−2^	8.94 × 10^−3^	8.94 × 10^−3^	3.78 × 10^−3^	[111]
Silicon-IT Solubility (mg/mL)	1.18 × 10^−1^	3.61 × 10^−1^	3.61 × 10^−1^	1.11 × 10	[111]
ESOL Solubility (mol/L)	2.41 × 10^−4^	3.13 × 10^−4^	3.13 × 10^−4^	3.72 × 10^−4^	[111]
Ali Solubility (mol/L)	8.44 × 10^−5^	2.90 × 10^−5^	2.90 × 10^−5^	9.72 × 10^−6^	[111]
Silicos-IT Solubility (mol/L)	5.16 × 10^−4^	1.17 × 10^−3^	1.17 × 10^−3^	2.86 × 10^−3^	[111]
ESOL Class	Soluble	Soluble	Soluble	Soluble	[111]
Ali Class	Moderately soluble	Moderately soluble	Moderately soluble	Moderately soluble	[111]
Silicos-IT class	Soluble	Soluble	Soluble	Soluble	[111]
Synthetic Accessibility	2.02	2.71	2.84	3.01	[111]
Bioavailability Score	0.55	0.56	0.56	0.11	[111,126]
Lipinski #violations	0	0	0	0	[111,117]
Ghose #violations	0	0	0	0	[111,118]
Veber #violations	0	0	0	1 (TPSA > 140)	[111,119]
Egan #violations	0	0	0	1 (TPSA > 131.6)	[111,120]
Muegge #violations	0	0	0	1 (TPSA > 150)	[111,121]
Leadlikeness #violations	1 (MW < 250)	0	0	1 (MW > 350)	[111,122]
PAINS #alerts	0	0	0	0	[111,123]
Brenk #alerts	1 (stilbene)	2 (stilbene, sufonic acid)	2 (stilbene, sulfonic acid)	2 (stilbene, sufonic acid)	[111,124]

The shadowed rows followed by white rows show the same parameters calculated by different methods.

**Figure 7:**
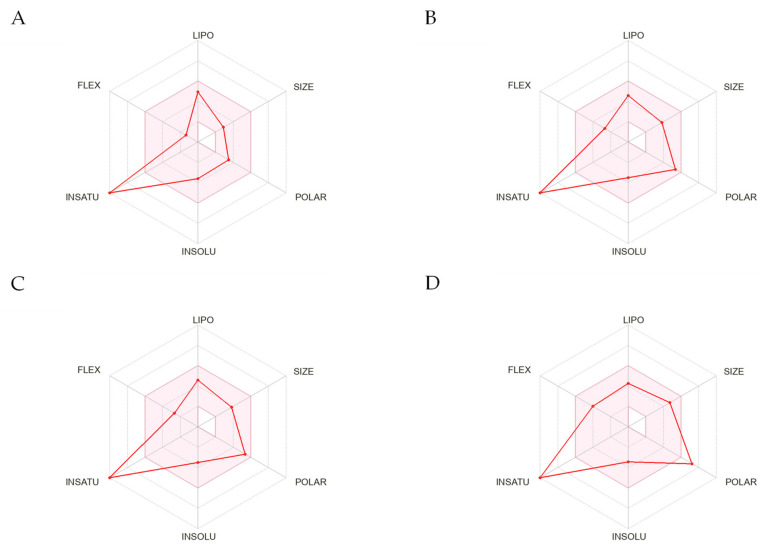
The Bioavailability Radar [111] for resveratrol (**A**), resveratrol-4′-*O*-sulfate (**B**), resveratrol-3-*O*-sulfate (**C**), resveratrol-3-*O*-4′-*O*-sulfate (**D**). The pink area represents the optimal range of parameters determining oral bioavailability. The pink has the following properties: LIPO—lipophilicity (XLOGP3 between −0.7 and +5.0), SIZE—molecular weight (MW between 150 and 500 g/mol), POLAR—polarity (TPSA between 20 and 130 Å^2^), INSOLU—solubility (log S not higher than 6), INSATU—saturation (fraction of carbons in the sp^3^ hybridisation not less than 0.25) [129] and FLEX - flexibility (no more than nine rotatable bonds.

Some in silico programs also provide the graphic presentation of predicted properties. The bioavailability has also been imaged by the Bioavailability Radar (Figure 7), which allows us to assess the parameters determining the compound’s bioavailability at a glance [111,127,128]. All tested compounds, resveratrol and its sulfates, were predicted not to be orally bioavailable because a fraction of carbons in the sp3 hybridisation is equal to zero in all these compounds, resveratrol-3-*O*-4′-*O*-sulfate is also too polar because its TPSA is 164.19.

## 4. Conclusions

The beneficial effects of resveratrol observed in lab experiments and as reported in numerous scientific publications are sometimes doubted due to the low bioavailability of resveratrol. This is because resveratrol is quickly metabolized in the intestinal wall and liver, leading to low intact molecule concentrations in the bloodstream. Scientific evidence suggests that the metabolites of resveratrol, such as sulfates and glucuronides, are not as biologically active as resveratrol itself. This raises questions about why the effects observed in lab experiments do not translate into the same level of activity in living organisms. However, there are indications that its metabolites may cause some of the effects of resveratrol in living organisms. Although the biological activity of these metabolites is relatively low, their effects may still be significant from a physiological point of view. Additionally, it is possible that in some cases, sulfatases in cells or intestinal bacteria may break down the sulfates, releasing resveratrol, which could directly affect cells and modulate intercellular signalling pathways. Examples of in silico predictions presented in this paper do not answer these questions definitively. Many of our calculations confirm the results of in vitro and in vivo experiments, indicating that resveratrol loses its biological activity after conjugation with sulfates. Sulfates were predicted as active only in a few predictions, including, surprisingly, androgen receptor binding. Although in silico methods, especially those used in this paper, are exciting, freely accessible, and easy to use to support in vitro and in vivo studies, these findings must still be validated with the results of classic laboratory studies. On the other hand, the development of in silico methods should be continued to create reliable tools for further research, especially in cases where compounds are challenging to synthesize, such as resveratrol metabolites.

## Figures and Tables

**Table 2:** The parameters describing the distribution, cellular uptake metabolism and interaction with receptors of resveratrol and its sulfates were calculated using freely available web-based programs [111,112,113,114]. References [112,113,114,115] correspond to SwissADME, admetSAR 2.0, PreADMET, and ProTox-II, respectively.

Assay	Resveratrol	Resveratrol-4′-*O*-Sulfate	Resveratrol-3-*O*-Sulfate	Resveratrol-3-*O*-4′-*O*-Sulfate	Ref.
CYP1A2 inhibitor	Yes	No	No	Yes	[111]
CYP1A2 inhibitor	Yes	No	No	No	[112]
CYP2C9 inhibitor	Yes	No	No	No	[111]
CYP2C9 inhibitor	Yes	Yes	Yes	Yes	[113]
CYP2C9 inhibitor	Yes	No	No	No	[112]
CYP2C19 inhibitor	No	No	No	No	[111]
CYP2C19 inhibitor	Yes	No	No	No	[113]
CYP2C19 inhibitor	Yes	No	No	No	[112]
CYP2D6 inhibitor	No	No	No	No	[111]
CYP2D6 inhibitor	No	No	No	No	[112]
CYP 2D6 inhibitor	No	No	No	No	[113]
CYP 3A4 inhibitor	Yes	Yes	Yes	No	[113]
CYP3A4 inhibitor	Yes	No	No	No	[111]
CYP3A4 inhibitor	Yes	No	No	No	[112]
CYP2C9 substrate	No	No	No	No	[112]
CYP2D6 substrate	No	No	No	No	[113]
CYP2D6 substrate	No	No	No	No	[112]
CYP3A4 substrate	No	No	No	No	[112]
CYP3A4 substrate	No	No	Weak	Weak	[113]
P-gp (MDR1, ABCB1) substrate	No	No	No	No	[111]
P-gp (MDR1, ABCB1) substrate	No	No	No	No	[112]
P-gp (MDR1, ABCB1) inhibitor	No	No	No	No	[113]
P-gp (MDR1, ABCB1) inhibitor	No	No	No	No	[112]
Aromatase binding	Yes	Yes	Yes	No	[112]
Aromatase	No	No	No	No	[114]
Aryl hydrocarbon Receptor (AhR)	No	No	No	No	[114]
CYP inhibitory promiscuity	Yes	No	No	No	[112]
OATP1B1 inhibitor	Yes	Yes	Yes	Yes	[112]
OATP1B3 inhibitor	Yes	Yes	Yes	Yes	[112]
OATP2B1 inhibitor	No	No	No	No	[112]
OCT1 inhibitor	No	No	No	No	[112]
OCT2 inhibitor	No	No	No	No	[112]
hERG_inhibition risk	medium	medium	Medium	low	[113]
BRCP inhibitor	No	No	No	No	[112]
BSEP (ABCB11) inhibitor	No	No	No	No	[112]
MATE1 inhibitor	No	No	No	No	[112]
Estrogen receptor binding	Yes	Yes	Yes	No	[112]
Estrogen Receptor Alpha (ER)	Yes	No	No	No	[114]
Androgen Receptor Ligand Binding Domain (AR-LBD)	No	No	No	No	[114]
Androgen Receptor (AR)	Yes	No	No	No	[114]
Androgen receptor binding	Yes	Yes	Yes	Yes	[112]
Estrogen Receptor Ligand Binding Domain (ER-LBD)	Yes	No	No	No	[114]
Glucocorticoid receptor binding	Yes	No	Yes	No	[112]
Thyroid receptor binding	Yes	No	No	No	[112]
Human Intestinal Absorption	Yes	Yes	Yes	Yes	[112]
Human oral bioavailability	No	No	No	No	[112]
Caco-2	Yes	Yes	Yes	No	[112]
GI absorption	High	High	High	Low	[111]
Blood Brain Barrier permeant	Yes	No	No	No	[111]
Blood Brain Barrier permeant	No	Yes	Yes	Yes	[112]
PPAR gamma	Yes	Yes	Yes	Yes	[112]
Human Ether-a-go-go-Related Gene inhibition	No	No	No	No	[112]
Heat shock factor response element (HSE)	No	No	No	No	[114]

The shadowed rows followed by white rows show the same parameters calculated by different programs.

**Table 3:** The basic toxicological parameters of resveratrol and its sulfates are calculated using free web-based softwares [112,113,114]. References [113,114,115] correspond to admetSAR 2.0, PreADMET, and ProTox-II, respectively.

Assay	Resveratrol	Resveratrol-4′-*O*-Sulfate	Resveratrol-3-*O*-Sulfate	Resveratrol-3-*O*-4′-*O*-Sulfate	Ref.
Eye corrosion	No	No	Yes	Yes	[112]
Eye irritation	Yes	Yes	Yes	Yes	[112]
Skin sensitisation	Yes	No	No	No	[112]
Fish aquatic toxicity	Yes	Yes	Yes	Yes	[112]
Crustacea aquatic toxicity	Yes	No	Yes	Yes	[112]
Honey bee toxicity	No	No	No	No	[112]
Avian toxicity	No	No	No	No	[112]
Nephrotoxicity	Yes	Yes	Yes	Yes	[112]
Hepatotoxicity	No	No	No	No	[112]
Respiratory toxicity	No	No	No	No	[112]
Hepatotoxicity	No	No	No	No	[114]
Immunotoxicity	No	No	No	No	[114]
Reproductive toxicity	No	No	No	No	[112]
Mutagenicity	No	No	No	No	[114]
Cytotoxicity	No	No	No	No	[114]
Mitochondrial toxicity	No	No	No	No	[112]
Mitochondrial Membrane Potential (MMP)	Yes	No	No	No	[114]
Micronuclear test	No	Yes	Yes	Yes	[112]
Ames test	Yes	No	Yes	No	[113]
Carcinogenicity mouse	No	Yes	No	Yes	[113]
Carcinogenicity rat	No	No	Yes	No	[113]
Carcinogenicity	No	No	No	No	[114]

## Data Availability

Not applicable.
